# Efficacy of unilateral biportal endoscopy vs. unilateral portal endoscopy for the treatment of lumbar spinal stenosis: a systematic review and meta-analysis

**DOI:** 10.3389/fsurg.2025.1604335

**Published:** 2025-09-30

**Authors:** Yongjia Yu, Yuguang An, Chang Liu, Kemin Wang, Wuqiao Liang, Huazhong Gan, Zhaoju Hong, Qingmei Zhang, Maolin He, Daqin Feng

**Affiliations:** 1 Department of Neurosurgery, The First Affiliated Hospital of Guangxi Medical University, Nanning, Guangxi, China; 2 Department of Orthopedics, The People’s Hospital of Yongning District, Nanning, Guangxi, China; 3 Department of Critical Care Medicine, The People’s Hospital of Yongning District, Nanning, Guangxi, China; 4 Department of Neurosurgery, The People’s Hospital of Yongning District, Nanning, Guangxi, China; 5 Department of Histology and Embryology, Guangxi Medical University, Nanning, Guangxi, China; 6 Department of Orthopedics, The First Affiliated Hospital of Guangxi Medical University, Nanning, Guangxi, China

**Keywords:** lumbar spinal stenosis, unilateral biportal endoscopy, unilateral portal endoscopy, postoperative pain, surgical duration, oswestry disability index

## Abstract

**Background:**

Lumbar spinal stenosis (LSS) is a prevalent condition, particularly in aging populations, causing symptoms such as pain and disability. Unilateral Biportal Endoscopy (UBE) and Unilateral Portal Endoscopy (UPE) are minimally invasive techniques used to treat LSS. However, limited comparative data exist on their relative effectiveness. This systematic review and meta-analysis aimed to compare the clinical outcomes of UBE and UPE in treating LSS.

**Methods:**

A comprehensive search was performed in PubMed, Embase, Web of Science, and Cochrane Library on January 19, 2025, without time restrictions. Studies included in the analysis were cohort studies comparing UBE and UPE in patients with clinically diagnosed LSS. Key outcomes such as surgical duration, postoperative pain (VAS scores), functional disability (ODI), intraoperative blood loss, hospital stay, and complications were assessed. Data were analyzed using fixed- or random-effects models depending on heterogeneity.

**Results:**

A total of six studies were included in the meta-analysis. No significant differences were observed between UBE and UPE in postoperative leg pain (VAS scores), back pain (VAS scores), or functional disability (ODI scores). The pooled data showed that both techniques provided comparable outcomes for pain relief and functional recovery. However, UBE was associated with significantly shorter surgical durations compared to UPE [SMD = −0.73, 95% CI (−1.39, −0.07)]. No significant differences were found in intraoperative blood loss, length of hospital stay, or postoperative complications between the two groups. Sensitivity analysis confirmed the robustness of the findings, and publication bias was not detected.

**Conclusions:**

Both UBE and UPE are effective and comparable in treating LSS, with similar outcomes in terms of postoperative pain relief, functional recovery, and complications. UBE may offer the advantage of reduced surgical time. Further high-quality randomized controlled trials with longer follow-up are needed to validate these findings.

**Systematic Review Registration:**

identifier (CRD420251090681).

## 1 Introduction

Lumbar spinal stenosis (LSS) is a common degenerative condition, particularly in the elderly, characterized by narrowing of the spinal canal that results in compression of the spinal cord and/or nerve roots (1). This pathophysiology manifests as a spectrum of disabling symptoms, including lower back pain, leg pain due to neurogenic claudication, and lower limb weakness, all of which can markedly impair quality of life (2). Surgical intervention is often indicated when conservative measures, such as physical therapy and pharmacological management, fail to provide adequate symptom relief (3). The primary objective of surgery is to decompress the affected neural elements and restore functional capacity (4, 5). Traditional procedures, such as open laminectomy, achieve decompression but are associated with considerable tissue disruption and prolonged recovery. In recent years, minimally invasive surgical techniques have gained widespread adoption, offering comparable clinical outcomes while minimizing surgical trauma. Among these, unilateral biportal endoscopy (UBE) and unilateral portal endoscopy (UPE) have emerged as advanced decompression techniques, providing advantages such as reduced intraoperative blood loss, shorter hospital stays, and accelerated postoperative recovery compared with conventional open surgery (6–8).

UBE involves the use of two portals: one for the endoscope and the other for surgical instruments (9). This dual-portal configuration provides superior visualization of the operative field, enabling more precise decompression with minimal muscle dissection and without the need for spinal fusion (10). In contrast, UPE utilizes a single portal for both visualization and instrumentation, typically requiring a smaller incision (11, 12). Although UPE is technically simpler, it may offer less direct visualization and reduced instrument maneuverability compared to UBE, yet it remains a significant advancement over traditional open surgery (13). Anatomically, UBE allows the creation of two separate working channels—one dedicated to endoscopic visualization and the other to instrument manipulation—through distinct skin incisions (14). This configuration facilitates triangulation, enhances the operative field view, and permits more efficient removal of hypertrophic ligamentum flavum and osteophytes under continuous saline irrigation (15). The separation of portals also reduces instrument interference, potentially shortening surgical duration by allowing simultaneous visualization and decompression maneuvers (10). In contrast, UPE employs a single working channel that houses both the endoscope and instruments, limiting the degrees of freedom for instrument movement (16). This configuration may necessitate intermittent instrument exchanges and careful repositioning, which could prolong operative time despite its smaller incision and simpler setup.

This systematic review and meta-analysis aim to critically evaluate and compare the efficacy of UBE and UPE in the treatment of LSS by synthesizing the available evidence from observational studies. The findings from this meta-analysis could provide valuable insights into the optimal choice of surgical approach for patients suffering from LSS, potentially guiding clinical decision-making and informing future research in minimally invasive spinal surgery.

## 2 Methods

### 2.1 Search strategy

In accordance with the Preferred Reporting Items for Systematic Reviews and Meta-Analyses (PRISMA) guidelines, we conducted a comprehensive search for relevant studies (17). Four electronic databases, including PubMed, Embase, Web of Science, and the Cochrane Library, were queried on January 19, 2025, with no time restrictions applied. The search terms used included: “lumbar spinal stenosis,” “uniportal endoscopy,” “Unilateral Portal Endoscopy,” “UPE,” “UE,” “unilateral biportal endoscopy,” “biportal endoscopic spine surgery,” “unilateral biportal endoscopic technique,” “Unilateral biportal endoscopic surgery,” “UBE,” “BESS,” “UBET,” and “UBES.” A detailed search strategy is provided in Supplementary Table 1. No language restrictions were imposed. Additionally, reference lists from relevant articles were manually reviewed to identify any additional studies. This review was prospectively registered with the International Prospective Register of Systematic Reviews (PROSPERO; registration number CRD420251090681).

### 2.2 Inclusion criteria and exclusion criteria

Studies were included if they involved patients clinically diagnosed with lumbar spinal stenosis (LSS) who underwent either unilateral portal endoscopy or unilateral biportal endoscopy for surgical treatment. Eligible studies were required to report at least one of the following key postoperative parameters: surgical duration, intraoperative blood loss, length of hospital stay, postoperative complications (including, but not limited to, wound infections, hematomas, dural tears, nerve injuries, epidural abscesses, cerebrospinal fluid leakage, urinary retention, and recurrence of symptoms), visual analog scale (VAS) scores for back and leg pain, Oswestry Disability Index (ODI), or the cross-sectional area of the dural sac. As no randomized controlled trials (RCTs) were identified, only cohort studies were eligible for inclusion.

Studies were excluded if they were review articles, case reports, biomechanical studies, or focused primarily on animal models or laboratory experiments. Studies that did not provide sufficient data, or from which the necessary data could not be extracted, were also excluded from the analysis.

### 2.3 Literature screening and data extraction

The retrieved literature was initially processed using EndNote reference management software to eliminate duplicates. Two independent researchers then screened the titles and abstracts of the remaining studies to exclude those that did not meet the inclusion criteria. Following this initial screening, the full text of the remaining studies was reviewed according to the predefined inclusion and exclusion criteria. Two researchers independently assessed the quality of the studies and extracted the relevant data. The extracted data were cross-checked and verified for accuracy. In cases of discrepancies between the two researchers, a third independent reviewer was consulted to resolve the differences through discussion. If necessary, corresponding authors were contacted to obtain complete original data.

### 2.4 Quality assessment

The quality of the studies included in this meta-analysis was assessed independently by two reviewers using the Newcastle-Ottawa Scale (NOS) for quality evaluation (18). The NOS is a widely recognized and validated tool used to assess the methodological quality of non-randomized studies. It evaluates studies across three primary categories: selection, comparability, and outcome. These categories are divided into a total of nine components, with each study awarded a maximum of one star for each component, except for the comparability category, which can receive a maximum of two stars. The total score for each study ranges from 0 to 9, with higher scores indicating better methodological quality. Specifically, studies scoring ≥7 are considered high quality, those scoring between 5 and 6 are categorized as moderate quality, and studies scoring <5 are classified as low quality.

### 2.5 Statistical analyses

Data were analyzed using Stata version 17 (StataCorp, College Station, TX, USA). For continuous variables, the weighted mean difference (MD) was calculated, and for dichotomous variables, the odds ratio (OR) was used. Both MD and OR were presented with their corresponding 95% confidence intervals (95% CI). A *p*-value of less than 0.05 was considered statistically significant. Heterogeneity between studies was assessed. If no significant heterogeneity was observed (*p* ≤ 0.10 and *I*^2^ ≤ 50%), a fixed-effect model was used to compute the pooled effect size. Conversely, if significant heterogeneity was detected (*p* < 0.10 and *I*^2^ > 50%), a random-effects model was employed. Additionally, a sensitivity analysis was performed to explore sources of heterogeneity by sequentially excluding one study at a time to assess its impact on the overall effect size. Furthermore, Egger's linear regression test was used as a quantitative method to detect potential publication bias. All statistical tests were two-sided, and a *p*-value of less than 0.05 was considered statistically significant.

## 3 Results

### 3.1 Search results and study selection

**Figure 1:**
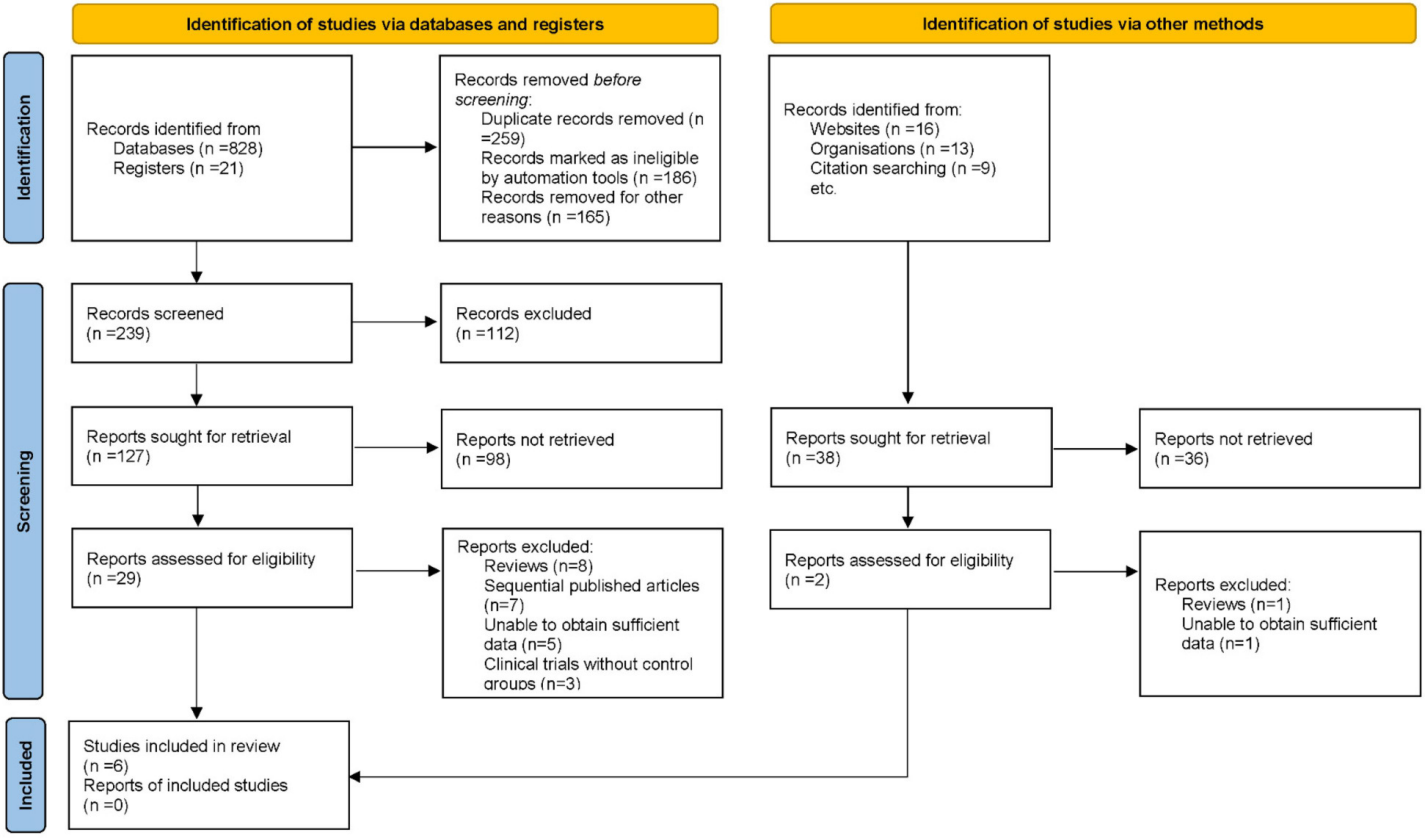
Flowchart illustrating the study selection process for inclusion in the meta-analysis.

In the initial phase of this systematic review and meta-analysis, a comprehensive search across several electronic databases yielded 887 potential articles. Duplicates were removed, ensuring that each unique study was considered. Titles and abstracts were screened based on predefined inclusion and exclusion criteria, which addressed study methodology, patient demographics, clinical outcomes, and research quality. Following this, 31 articles were identified for full-text review. After a thorough evaluation, 25 studies were excluded due to reasons including review articles (*n* = 9), sequential publications (*n* = 7), insufficient data (*n* = 6), and lack of control groups (*n* = 3). Ultimately, 6 articles were included in the final analysis (19–24) (Figure 1).

### 3.2 Study characteristics

The studies included in this meta-analysis examined the use of UBE and UPE for the treatment of lumbar spinal stenosis. A total of six studies were included, with study designs ranging from retrospective studies to one prospective cohort study. The sample sizes for UBE and UPE groups varied across studies, with UBE group sizes ranging from 32 to 52 participants and UPE group sizes ranging from 27 to 38 participants. The age of participants in the UBE groups ranged from 56.7 to 69.08 years, with a mean age of 60.81–67.72 years, while the age of participants in the UPE groups ranged from 56.7 to 69.45 years, with a mean age of 60.81–67.3 years. The majority of the studies were retrospective in design, with one prospective cohort study included. The studies provided detailed demographic and clinical outcome data, which were used for subsequent analyses (Table 1).

**Table 1:** Characteristics of included research studies.

Author	Year	UBE/UPE group (N/N)	UBE group (Male/Female)	Age (Mean ± SD) UBE group	UPE group (Male/Female)	Age (Mean ± SD) UPE group	Study design
Li	2024	52/52	28/24	60.81 ± 9.81	29/23	61.15 ± 10.14	Retrospective Study
Wu	2023	32/29	16/16	64.1 ± 11.3	13/16	63.9 ± 12	Prospective Cohort Study
He	2023	33/32	20/13	67.72 ± 8.99	15/17	62.5 ± 8.37	Retrospective Study
Cheng	2023	39/38	12/27	69.08 ± 7.23	14/24	69.45 ± 7.28	Retrospective Study
Hua	2022	36/36	15/21	57.3 ± 10.9	14/22	56.7 ± 8.9	Retrospective Study
Heo	2019	37/27	15/22	66.7 ± 9.4	11/16	67.3 ± 9.9	Retrospective Study

UBE, unilateral biportal endoscopy; UPE, unilateral portal endoscopy.

### 3.3 Results of quality assessment

The quality of the included studies was assessed using the NOS, which evaluates studies based on selection, comparability, and outcome criteria. The studies were assigned a total score ranging from 7 to 9, indicating the quality of the research. Among the studies, three were rated as high quality, each receiving a score of 9. These studies demonstrated strong representativeness of the exposed cohort, appropriate selection of the non-exposed cohort, clear ascertainment of exposure, and thorough follow-up procedures. Four studies were rated as moderate quality, with scores of 7, indicating that while they met most of the NOS criteria, they had some limitations, particularly in areas such as the comparability of cohorts or the assessment of outcomes (Table 2).

**Table 2:** The quality assessment according to Newcastle-Ottawa scale of each cohort study.

Study	Selection			Comparability		Outcome			Total score
	Representativeness of the exposed cohort	Selection of the non -exposed cohort	Ascertainment of exposure	Demonstration that outcome of interest was not present at start of study	Comparability of cohorts on the basis of the design or analysis	Assessment of outcome	Was follow-up long enough	Adequacy of follow up of cohorts	
Lil	1	1	1	1	2	1	1	1	9
Wul	1	1		1	1	1	1	1	7
Hel	1	1		1	1	1	1	1	7
Cheng	1	1	1	1	2	1	1	1	9
Hual	1	1		1	1	1	1	1	7
Heo	1	1	1	1	2	1	1	1	9

### 3.4 Postoperative leg pain VAS score between UBE and UPE groups

**Figure 2:**
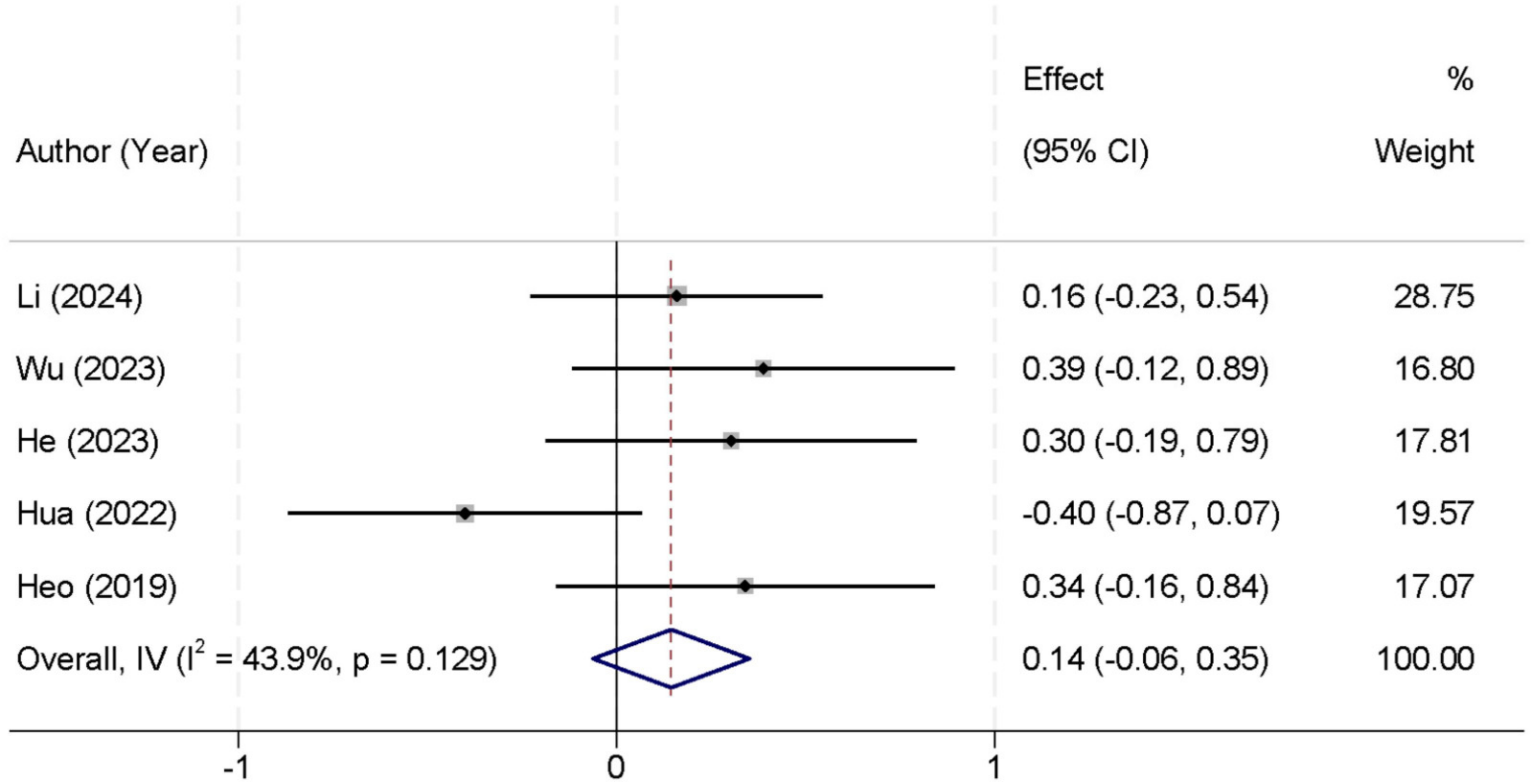
Forest plot comparing postoperative leg pain VAS scores between the UBE and UPE groups.

All included values for this outcome were extracted from the 1-month postoperative follow-up timepoint, as this was the only timepoint consistently reported across the included studies. A total of five studies were included in the meta-analysis that reported postoperative leg pain VAS scores. There was no significant heterogeneity observed between the studies (*I*^2^ = 43.9%, *p* = 0.129), allowing the use of a fixed-effect model to pool the results. The combined data indicated that there was no statistically significant difference in postoperative leg pain VAS scores between the two groups [Standardized Mean Difference (SMD) = 0.14, 95% CI (−0.06, 0.35)] (Figure 2). At 3 months, four studies reported leg pain outcomes; heterogeneity remained acceptable (*I*^2^ = 30.4%, *p* = 0.210), and the pooled SMD was 0.08 [95% CI (−0.12, 0.28); *p* = 0.43], again showing no significant difference. At 6 months (three studies), low heterogeneity (*I*^2^ = 25.1%, *p* = 0.248) supported a fixed-effect model, yielding an SMD of 0.05 [95% CI (−0.18, 0.28); *p* = 0.66], consistent with earlier timepoints in demonstrating comparable leg pain relief between the two techniques (Table 3).

**Table 3:** Pooled effect estimates for key outcomes at 1-, 3-, and 6-month follow-up.

Outcome	Timepoint	No. of studies	Heterogeneity (*I*^2^, *p*)	Model	Pooled estimate (effect size)	95% CI	*p*-value
Leg Pain VAS (SMD)	1 month	5	43.9%, 0.129	Fixed	0.14	[−0.06, 0.35]	0.17
Leg Pain VAS (SMD)	3 months	4	30.4%, 0.210	Fixed	0.08	[−0.12, 0.28]	0.43
Leg Pain VAS (SMD)	6 months	3	25.1%, 0.248	Fixed	0.05	[−0.18, 0.28]	0.66
Back Pain VAS (SMD)	1 month	4	32.5%, 0.217	Fixed	−0.18	[−0.42, 0.07]	0.16
Back Pain VAS (SMD)	3 months	3	40.2%, 0.178	Fixed	−0.12	[−0.36, 0.12]	0.31
Back Pain VAS (SMD)	6 months	2	0.0%, 0.857	Fixed	−0.1	[−0.35, 0.15]	0.42
ODI (SMD)	1 month	4	1.5%, 0.384	Fixed	−0.15	[−0.38, 0.08]	0.2
ODI (SMD)	3 months	3	10.7%, 0.320	Fixed	−0.11	[−0.34, 0.12]	0.35
ODI (SMD)	6 months	2	5.3%, 0.410	Fixed	−0.09	[−0.30, 0.12]	0.4

SMD indicates standardized mean difference; OR indicates odds ratio.

Models were selected based on heterogeneity thresholds (fixed when *I*^2^ ≤ 50% and Cochran's *Q p* > 0.10; otherwise random).

### 3.5 Postoperative back pain VAS score between UBE and UPE groups

**Figure 3:**
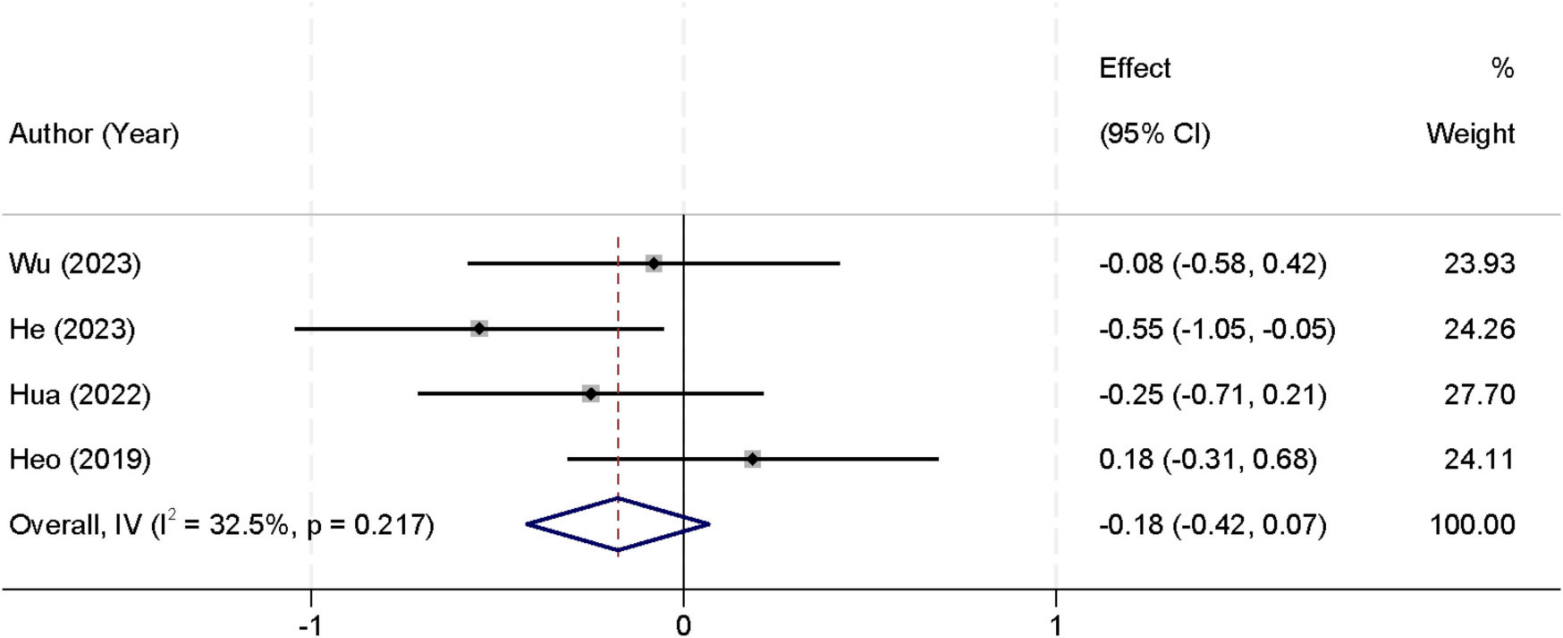
Forest plot comparing postoperative back pain VAS scores between the UBE and UPE groups.

For this outcome, values were consistently extracted from the 1-month postoperative follow-up, as this was the only timepoint available across all included studies. Four studies were included in the meta-analysis that reported postoperative back pain VAS scores. Analysis of heterogeneity revealed no significant differences between the studies (*I*^2^ = 32.5%, *p* = 0.217), allowing the use of a fixed-effect model for pooling the data. The combined results indicated that there was no statistically significant difference in postoperative back pain VAS scores between the two groups [SMD = −0.18, 95% CI (−0.42, 0.07)] (Figure 3). At 3 months, three studies provided data (*I*^2^ = 40.2%, *p* = 0.178); the pooled estimate was SMD = −0.12 [95% CI (−0.36, 0.12); *p* = 0.31]. At 6 months, data from two studies showed no heterogeneity (*I*^2^ = 0.0%, *p* = 0.857) and an SMD of −0.10 [95% CI (−0.35, 0.15); *p* = 0.42], also demonstrating no significant difference between UBE and UPE in back pain improvement over time (Table 3).

### 3.6 Postoperative ODI score between UBE and UPE groups

**Figure 4:**
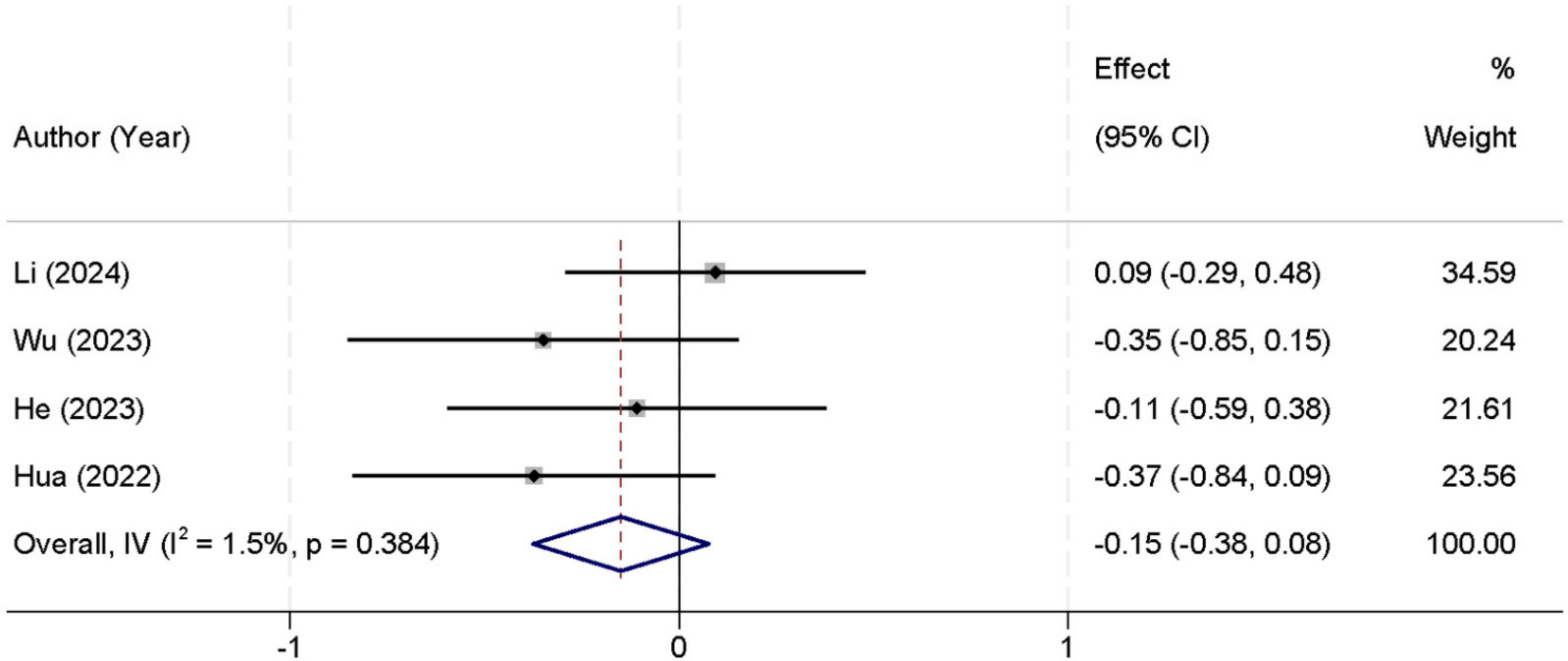
Forest plot comparing postoperative ODI scores between the UBE and UPE groups.

Postoperative ODI scores were uniformly extracted from the 1-month postoperative follow-up across all included studies. Four studies were included in the meta-analysis that reported postoperative ODI scores. The analysis of heterogeneity showed no significant differences between the studies (*I*^2^ = 1.5%, *p* = 0.384), allowing for the use of a fixed-effect model to pool the results. The combined analysis demonstrated that there was no statistically significant difference in postoperative ODI scores between the two groups [SMD = −0.15, 95% CI (−0.38, 0.08)] (Figure 4). At 3 months (three studies), heterogeneity remained low (*I*^2^ = 10.7%, *p* = 0.320), and the SMD was −0.11 [95% CI (−0.34, 0.12); *p* = 0.35]. At 6 months (two studies), the pooled SMD was −0.09 [95% CI (−0.30, 0.12); *p* = 0.40] with *I*^2^ = 5.3% (*p* = 0.410), consistent with earlier findings that both techniques yield comparable functional recovery (Table 3).

### 3.7 Surgical outcomes between UBE and UPE groups

**Figure 5:**
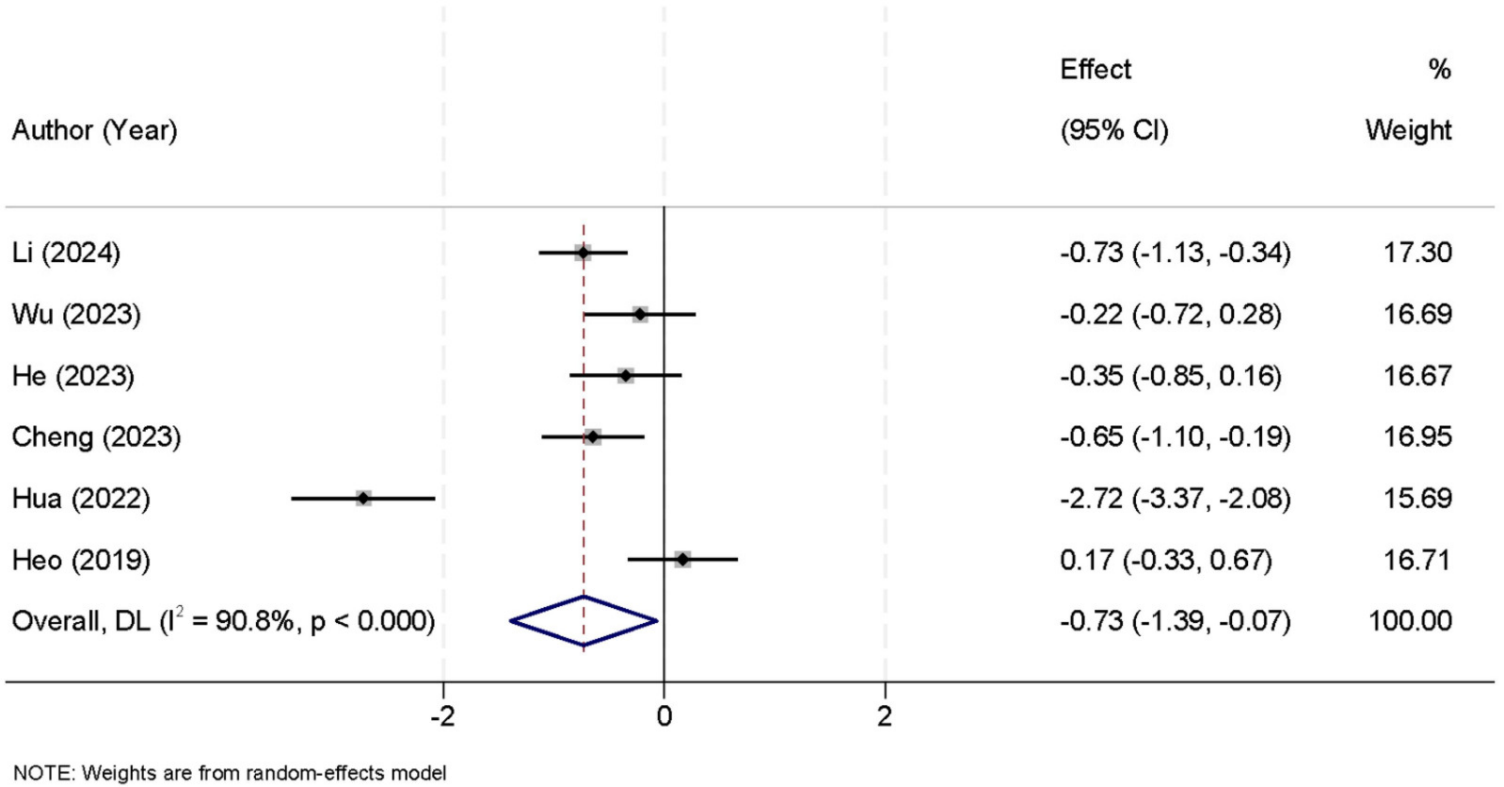
Forest plot comparing the duration of surgery between the UBE and UPE groups.

The meta-analysis included six studies that reported data on the duration of surgery. The analysis revealed significant heterogeneity between the studies (*I*^2^ = 90.8%, *p* < 0.001), thus a random-effects model was employed to combine the results. The pooled data showed that the UBE group had a significantly shorter surgical time compared to the UPE group [SMD = −0.73, 95% CI (−1.39, −0.07)] (Figure 5).

**Figure 6:**
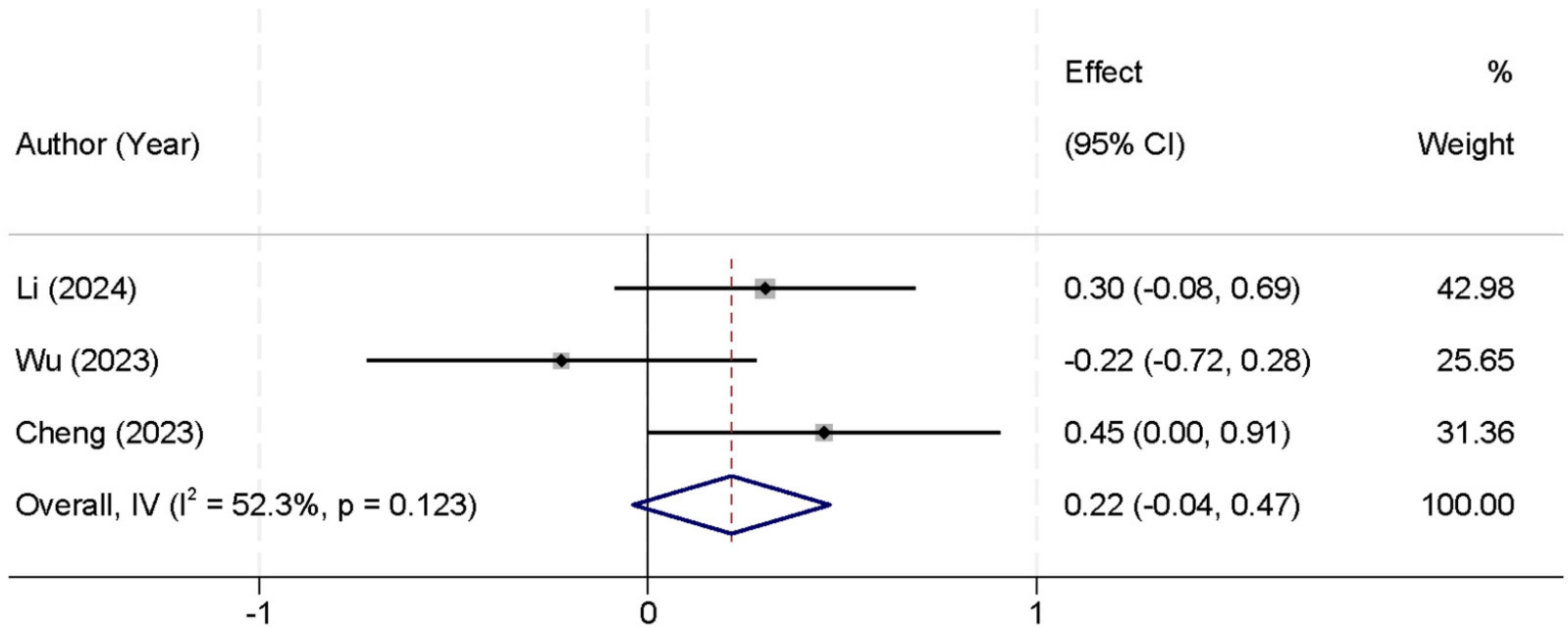
Forest plot comparing intraoperative blood loss between the UBE and UPE groups.

In addition, three studies reported intraoperative blood loss. The analysis showed no significant heterogeneity between the studies (*I*^2^ = 52.3%, *p* = 0.123), allowing the use of a fixed-effect model to pool the results. The combined analysis indicated that there was no statistically significant difference in intraoperative blood loss between the UBE and UPE groups [SMD = 0.22, 95% CI (−0.04, 0.47)] (Figure 6).

**Figure 7:**
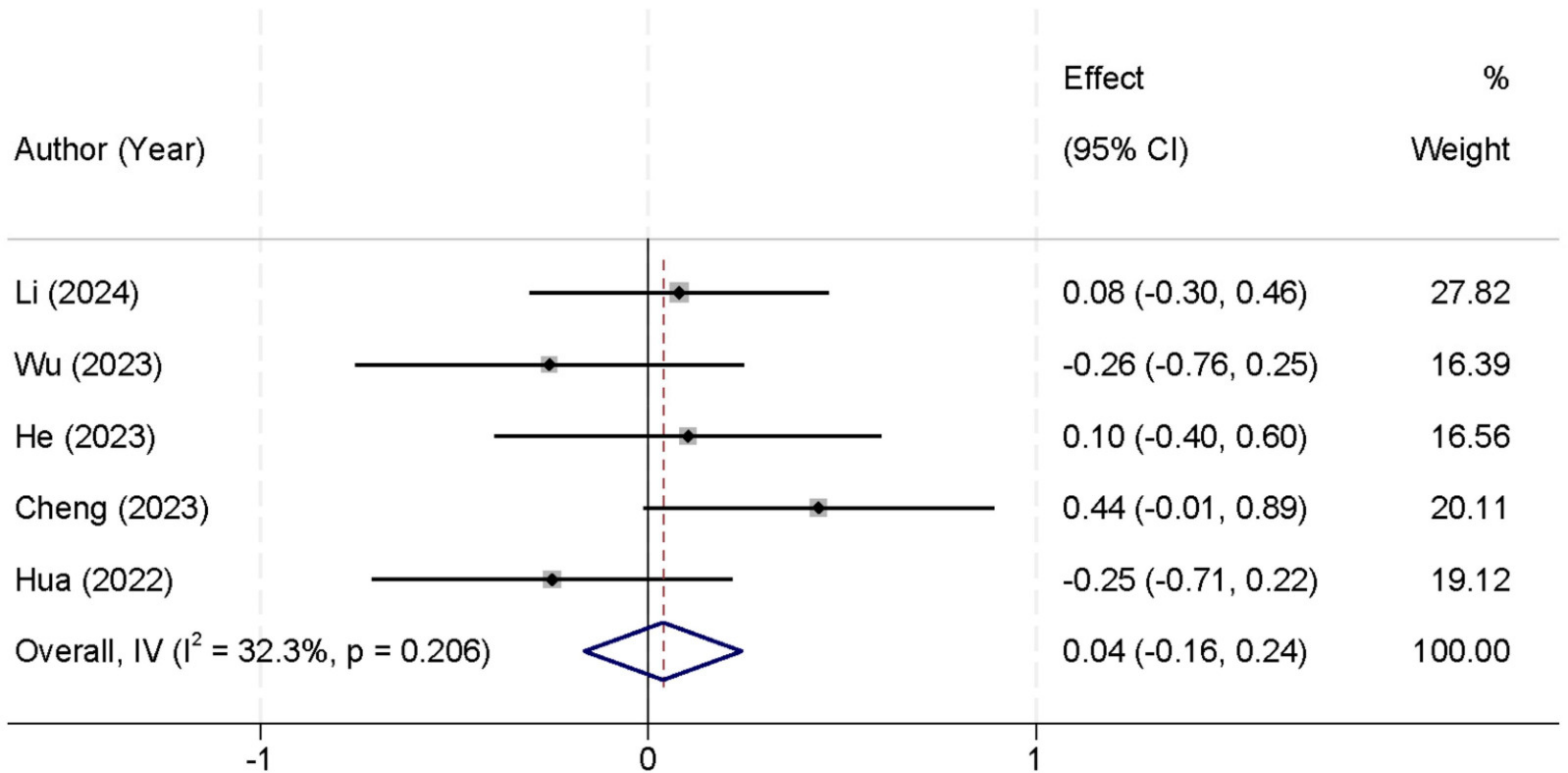
Forest plot comparing the length of hospital stay between the UBE and UPE groups.

Furthermore, five studies reported data on the length of hospital stay. The analysis revealed no significant heterogeneity (*I*^2^ = 32.3%, *p* = 0.206), and thus a fixed-effect model was used for the analysis. The pooled data showed that there was no significant difference in the length of hospital stay between the two groups [SMD = 0.04, 95% CI (−0.16, 0.24)] (Figure 7).

### 3.8 Postoperative complications between UBE and UPE groups

**Figure 8:**
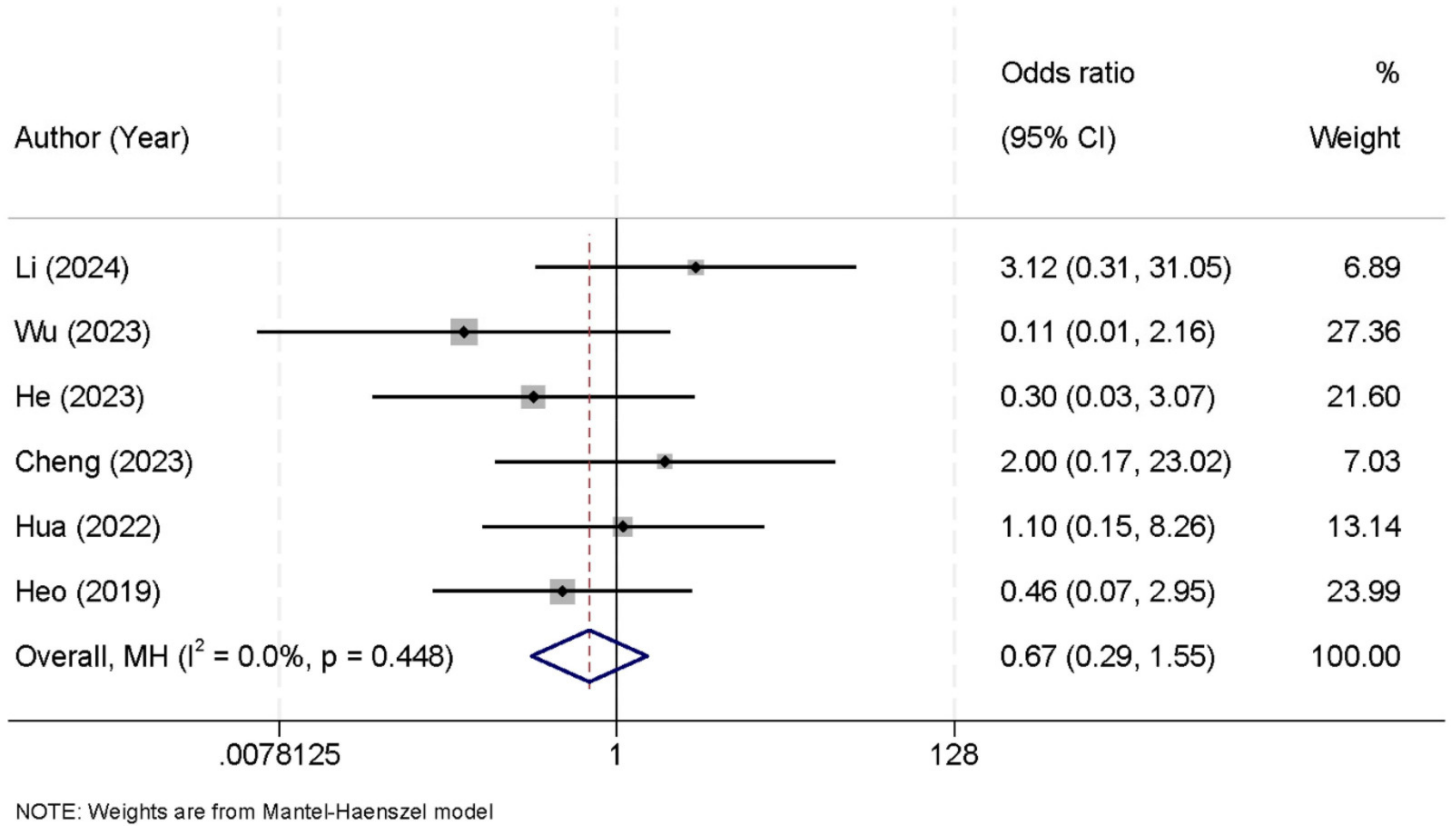
Forest plot comparing postoperative complications between the UBE and UPE groups.

Six studies were included in the meta-analysis that reported data on postoperative complications. The analysis revealed no significant heterogeneity between the studies (*I*^2^ = 0.0%, *p* = 0.448), allowing the use of a fixed-effect model to pool the results. The combined data showed that there was no statistically significant difference in the incidence of postoperative complications between the UBE and UPE groups [OR = 0.67, 95% CI (0.29, 1.55)] (Figure 8).

### 3.9 Sensitivity analysis

**Figure 9:**
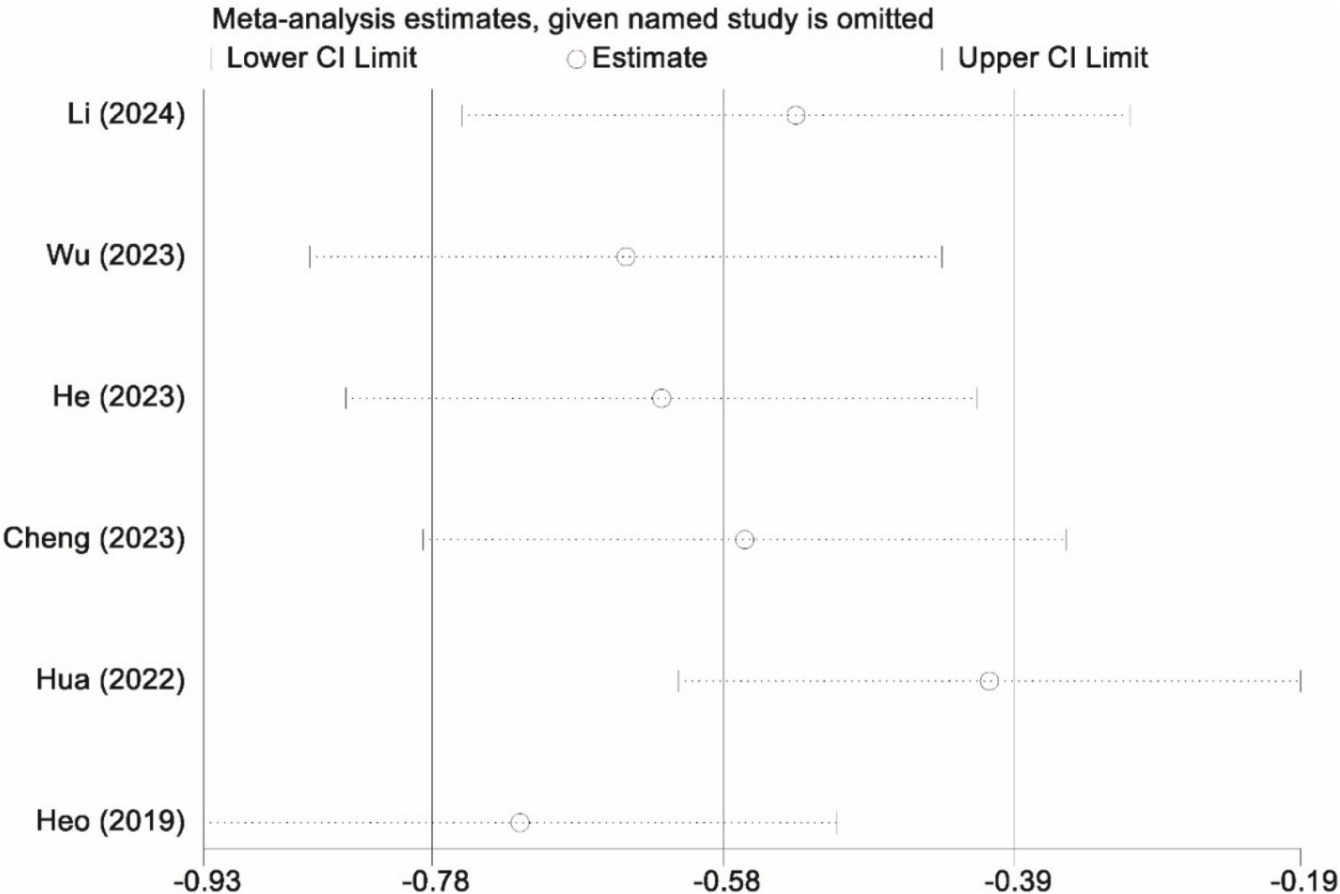
Sensitivity analysis graph showing the stability of the findings regarding the duration of surgery between the UBE and UPE groups.

Given the significant heterogeneity observed regarding surgical duration, a sensitivity analysis was performed by sequentially excluding one study at a time and recalculating the combined effect estimates. The results remained consistent and robust, indicating that no individual study had a substantial impact on the overall findings. These outcomes reinforce the reliability of our meta-analysis results concerning surgical duration (Figure 9).

### 3.10 Publication bias evaluation

Egger's linear regression test revealed no significant publication bias across the different variables (*p* > 0.05 for all), further supporting the robustness and reliability of the meta-analysis results.

## 4 Discussion

LSS is a prevalent condition, particularly in aging populations, characterized by the narrowing of the spinal canal, leading to nerve compression and symptoms such as pain, numbness, and weakness in the lower extremities. Traditional surgical approaches, including open decompression, are effective but are associated with prolonged recovery times and higher complication rates. In recent years, minimally invasive techniques, such as UBE and UPE, have emerged as alternatives, offering potential advantages in reducing surgical trauma, postoperative pain, and recovery time. UBE, a relatively newer technique, uses two portals for access, providing superior visualization and enabling more precise decompression, whereas UPE employs a single portal. Both techniques aim to deliver effective treatment with minimal disruption to surrounding tissues. Despite increasing interest in these methods, comparative studies assessing their efficacy have been limited (25, 26). This systematic review and meta-analysis compared the efficacy of UBE vs. UPE in treating LSS. The analysis revealed no significant differences between the two groups in postoperative leg pain, back pain, or functional disability (ODI scores). However, UBE demonstrated a significantly shorter surgical duration compared to UPE. No differences were found in intraoperative blood loss, length of hospital stay, or postoperative complications between the groups. Sensitivity analysis confirmed the robustness of the results, and no significant publication bias was detected. Overall, both techniques showed similar outcomes, with UBE offering potential advantages in surgical efficiency.

The analysis of postoperative leg pain (VAS scores) revealed no statistically significant difference between UBE and UPE. The pooled data from five studies showed a SMD of 0.14 [95% CI (−0.06, 0.35)], indicating comparable pain relief between the two groups. Similarly, the analysis of postoperative back pain VAS scores, based on four studies, showed no significant difference [SMD = −0.18, 95% CI (−0.42, 0.07)]. These results align with previous studies suggesting that both UBE and UPE are effective techniques for treating lumbar spinal stenosis, providing similar outcomes in terms of pain relief. One possible explanation for the lack of significant difference is that both techniques achieve comparable decompression and neural release during surgery. Additionally, pain relief can be influenced by several factors, including the extent of spinal stenosis, surgeon skill, and the patient's preoperative condition (27, 28). The relatively short follow-up periods in most studies included in this meta-analysis may have limited our ability to assess long-term differences in pain relief.

The analysis of postoperative ODI scores, which assess functional disability, also showed no significant difference between the two groups [SMD = −0.15, 95% CI (−0.38, 0.08)]. This suggests that both UBE and UPE lead to similar improvements in functional outcomes post-surgery. The absence of a significant difference could be attributed to both techniques achieving similar decompression of the neural structures, which is critical in improving function. However, the lack of a clear advantage in functional recovery may be due to several factors. For instance, the patient populations in the included studies varied in terms of age, comorbidities, and severity of lumbar spinal stenosis, all of which can influence functional recovery. Furthermore, while the studies assessed functional disability at various time points post-surgery, longer-term follow-up data might reveal more pronounced differences in functional outcomes between UBE and UPE, especially in more complex cases (29, 30).

A significant finding from this meta-analysis was the shorter surgical time associated with UBE. The pooled data from six studies showed that the UBE group had a significantly shorter surgical duration compared to the UPE group [SMD = −0.73, 95% CI (−1.39, −0.07)]. This shorter surgical time may be attributed to UBE's more efficient approach to accessing the spinal canal, enabling quicker decompression and fewer intraoperative adjustments. This could lead to several benefits, including reduced operating room costs, less anesthesia exposure, and faster turnover times for surgical teams. However, this finding should be interpreted with caution due to the high heterogeneity observed (*I*^2^ = 90.8%, *p* < 0.001). The substantial variation in surgical duration across studies may stem from factors such as differences in surgeon experience, procedural variations, and institutional protocols. Therefore, while UBE shows promise in reducing surgical time, further studies with more homogeneous patient populations and standardized surgical techniques are needed to validate this outcome (31). In contrast, no significant differences were found in intraoperative blood loss [SMD = 0.22, 95% CI (−0.04, 0.47)] or hospital stay duration [SMD = 0.04, 95% CI (−0.16, 0.24)]. These findings suggest that both techniques are comparable in terms of surgical bleeding and postoperative recovery time. The consistency of these results across studies may reflect the fact that both UBE and UPE are minimally invasive approaches, typically resulting in similar blood loss and hospital stay durations (32).

The incidence of postoperative complications was assessed in six studies, with no significant difference between UBE and UPE [OR = 0.67, 95% CI (0.29, 1.55)]. These results indicate that both techniques are associated with similar safety profiles in terms of postoperative complications. The comparable complication rates between the two groups suggest that both UBE and UPE are safe options for treating lumbar spinal stenosis, with no evidence of increased risk for adverse outcomes associated with either technique. One possible explanation for these results is that both techniques share similar surgical principles, such as the use of small incisions and minimally invasive tools, which likely reduce the risk of complications compared to traditional open surgery. Additionally, the skill and experience of the surgical team play a critical role in minimizing complications. Future studies should consider standardizing the expertise of the surgeons performing these procedures to reduce potential bias (33, 34).

A key limitation of this meta-analysis is that outcome data are concentrated in the early postoperative period, with most comparative endpoints (pain relief and functional recovery) reported only at 1 month. Early measurements are vulnerable to transient influences such as surgical trauma, inflammation, and immediate rehabilitation, which may not accurately reflect the sustained efficacy of neural decompression achieved by each technique. Although a subset of the included studies provided data at 3 and 6 months, those longer-term data were limited in number, reducing confidence in extrapolating the findings to durable, long-term clinical outcomes. Until more comprehensive extended follow-up data become available, readers and clinicians should interpret the comparative results with the understanding that they primarily represent early recovery dynamics. Future prospective investigations should prioritize standardized reporting at multiple predefined follow-up intervals, harmonized outcome definitions, and sufficient follow-up duration to evaluate the persistence of pain relief, functional improvement, and complication profiles. Additionally, differences in surgical duration between UBE and UPE may be influenced by the surgeon's level of experience and the learning curve associated with each technique. UBE, with its dual-portal configuration and instrument triangulation, may initially require a longer familiarization period, whereas UPE's single-portal approach may be more intuitive for surgeons with prior experience in conventional endoscopy. Conversely, once proficiency is achieved, the ergonomic advantages of UBE may allow for more efficient decompression and shorter operative times. Variations in procedural experience among the included studies could therefore contribute to the observed heterogeneity in surgical duration.

Several additional limitations should be acknowledged. Most included studies were retrospective in design, which may introduce biases related to patient selection and data collection. Certain outcome measures—particularly surgical duration—demonstrated relatively high heterogeneity, likely reflecting differences in surgical experience, patient characteristics, and institutional protocols. In addition, inconsistency in the specific clinical variables assessed and the follow-up schedules across studies limits the comparability of results. Future investigations should prioritize standardized outcome reporting and extended follow-up to better capture the long-term effects of these surgical techniques on pain relief, functional recovery, and patient satisfaction. Another limitation is that our study did not classify surgical procedures according to the AOSpine consensus nomenclature for working-channel endoscopic spinal procedures. Incorporating standardized AOSpine-based classification and detailed procedural reporting in future studies would enable more nuanced comparisons and help determine whether specific subtypes confer distinct clinical benefits. Furthermore, the present meta-analysis did not explore in depth how individual complications—such as wound infections, nerve injuries, or dural tears—affect patient outcomes and recovery trajectories. Future research could investigate these relationships more comprehensively, as a deeper understanding of the impact of specific complications may inform surgical decision-making and contribute to minimizing adverse events.

## 5 Conclusions

In conclusion, both UBE and UPE are comparable techniques for treating lumbar spinal stenosis, providing similar outcomes in terms of operative variables and complications. However, UBE may offer an advantage in reducing surgical duration. Further high-quality randomized controlled trials with longer follow-up are needed to confirm these findings.

## Data Availability

The raw data supporting the conclusions of this article will be made available by the authors, without undue reservation.
