# Supplementary material for: Efficacy of unilateral biportal endoscopy vs. unilateral portal endoscopy for the treatment of lumbar spinal stenosis: a systematic review and meta-analysis
Source: Front Surg. 2025 Sep 30;12:1604335. doi: 10.3389/fsurg.2025.1604335 (PMC12518314; doi:10.3389/fsurg.2025.1604335)
Supplement: Supplementary file 1 [file Table1.docx]

Supplementary Table I Search Strategy for Four Databases.

| Database | Search Strategy |
| --- | --- |
| PubMed | ("lumbar spinal stenosis"[MeSH Terms] OR "lumbar spinal stenosis"[Title/Abstract]) AND ("uniportal endoscopy"[Title/Abstract] OR "Unilateral Portal Endoscopy"[Title/Abstract] OR "UPE"[Title/Abstract] OR "UE"[Title/Abstract] OR "unilateral biportal endoscopy"[Title/Abstract] OR "biportal endoscopic spine surgery"[Title/Abstract] OR "unilateral biportal endoscopic technique"[Title/Abstract] OR "Unilateral biportal endoscopic surgery"[Title/Abstract] OR "UBE"[Title/Abstract] OR "BESS"[Title/Abstract] OR "UBET"[Title/Abstract] OR "UBES"[Title/Abstract]) AND ("Surgical outcomes"[MeSH Terms] OR "clinical outcomes"[MeSH Terms] OR "efficacy"[Title/Abstract] OR "treatment outcome"[MeSH Terms] OR "complications"[Title/Abstract] OR "patient satisfaction"[Title/Abstract]) AND ("Systematic review"[Publication Type] OR "Meta-analysis"[Publication Type] OR "Randomized controlled trial"[Publication Type] OR "Cohort study"[Publication Type] OR "Observational study"[Publication Type]) |
| Embase | ("lumbar spinal stenosis" OR "lumbar stenosis" OR "lumbar spinal canal stenosis") AND ("uniportal endoscopy" OR "Unilateral Portal Endoscopy" OR "UPE" OR "UE" OR "unilateral biportal endoscopy" OR "biportal endoscopic spine surgery" OR "unilateral biportal endoscopic technique" OR "Unilateral biportal endoscopic surgery" OR "UBE" OR "BESS" OR "UBET" OR "UBES") AND ("surgical outcomes" OR "clinical outcomes" OR "efficacy" OR "treatment outcome" OR "complications" OR "patient satisfaction") AND ("systematic review" OR "meta-analysis" OR "randomized controlled trial" OR "cohort study" OR "observational study") |
| Web of Science | ("lumbar spinal stenosis" OR "lumbar stenosis" OR "lumbar spinal canal stenosis") AND ("uniportal endoscopy" OR "Unilateral Portal Endoscopy" OR "UPE" OR "UE" OR "unilateral biportal endoscopy" OR "biportal endoscopic spine surgery" OR "unilateral biportal endoscopic technique" OR "Unilateral biportal endoscopic surgery" OR "UBE" OR "BESS" OR "UBET" OR "UBES") AND ("surgical outcomes" OR "clinical outcomes" OR "efficacy" OR "treatment outcome" OR "complications" OR "patient satisfaction") AND ("systematic review" OR "meta-analysis" OR "randomized controlled trial" OR "cohort study" OR "observational study") |
| Cochrane Library | ("lumbar spinal stenosis" OR "lumbar stenosis" OR "lumbar spinal canal stenosis") AND ("uniportal endoscopy" OR "Unilateral Portal Endoscopy" OR "UPE" OR "UE" OR "unilateral biportal endoscopy" OR "biportal endoscopic spine surgery" OR "unilateral biportal endoscopic technique" OR "Unilateral biportal endoscopic surgery" OR "UBE" OR "BESS" OR "UBET" OR "UBES") AND ("surgical outcomes" OR "clinical outcomes" OR "efficacy" OR "treatment outcome" OR "complications" OR "patient satisfaction") AND ("systematic review" OR "meta-analysis" OR "randomized controlled trial" OR "cohort study" OR "observational study") |
